# Randomized Blinded Placebo-Controlled Trials of Renal Sympathetic Denervation for Hypertension: A Meta-Analysis

**DOI:** 10.1016/j.carrev.2021.01.031

**Published:** 2022-01

**Authors:** Yousif Ahmad, Christopher Kane, Ahran D. Arnold, Christopher M. Cook, Daniel Keene, Matthew Shun-Shin, Graham Cole, Rasha Al-Lamee, Darrel P. Francis, James P. Howard

**Affiliations:** a National Heart and Lung Institute, Imperial College London, London, United Kingdom; b Smidt Heart Institute, Cedars Sinai Medical Center, Los Angeles, USA

**Keywords:** Hypertension, Renal denervation, Meta-analysis

## Abstract

**Background:**

The efficacy of renal denervation has been controversial, but the procedure has now undergone several placebo-controlled trials. New placebo-controlled trial data has recently emerged, with longer follow-up of one trial and the full report of another trial (which constitutes 27% of the total placebo-controlled trial data). We therefore sought to evaluate the effect of renal denervation on ambulatory and office blood pressures in patients with hypertension.

**Methods:**

We systematically identified all blinded placebo-controlled randomized trials of catheter-based renal denervation for hypertension. The primary efficacy outcome was ambulatory systolic blood pressure change relative to placebo. A random-effects meta-analysis was performed.

**Results:**

6 studies randomizing 1232 patients were eligible. 713 patients were randomized to renal denervation and 519 to placebo. Renal denervation significantly reduced ambulatory systolic blood pressure (−3.52 mmHg; 95% CI −4.94 to −2.09; *p* < 0.0001), ambulatory diastolic blood pressure (−1.93 mmHg; 95% CI −3.04 to −0.83, *p* = 0.0006), office systolic blood pressure size (−5.10 mmHg; 95% CI −7.31 to −2.90, *p* < 0.0001) and office diastolic pressure (effect size −3.11 mmHg; 95% CI −4.43 to −1.78, p < 0.0001). Adverse events were rare and not more common with denervation.

**Conclusions:**

The totality of blinded, randomized placebo-controlled data shows that renal denervation is safe and provides genuine reduction in blood pressure for at least 6 months post-procedure. If this effect continues in the long term, renal denervation might provide a life-long 10% relative risk reduction in major adverse cardiac events and 7.5% relative risk reduction in all-cause mortality.

## Highlights

• In this updated meta-analysis of 6 trials and 1232 patients, renal denervation reduced ambulatory systolic, ambulatory diastolic, office systolic, and office diastolic blood pressure.
• The totality of blinded, randomized placebo-controlled data shows that renal denervation is safe and provides genuine reduction in blood pressure for at least 6 months post-procedure.
• If this effect continues in the long term, renal denervation might provide a life-long 10% relative risk reduction in major adverse cardiac events and 7.5% relative risk reduction in all-cause mortality.

## 1 Introduction

Renal denervation (RDN) was introduced as a procedure to lower blood pressure (BP). Early trials of RDN were unblinded and showed reductions in office blood pressure (OBP) of ~30 mmHg [1]. However, the first blinded trial of RDN, Symplicity HTN-3, elicited surprise when it reported a non-significant reduction of only 2.4 mmHg versus placebo [2].

RDN has now undergoing several placebo-controlled trials, and meta-analysis of these trials has shown significantly reduced ambulatory and office systolic BP compared with placebo [3]. However, the total number of patients randomized in placebo-controlled trials has been small. New placebo-controlled trial data has recently emerged, with longer follow-up of one trial [4] and the full report of another trial [5] (which constitutes 27% of the total placebo-controlled trial data).

We therefore conducted an updated meta-analysis of RDN for hypertension, including the totality of randomized placebo-controlled trial data now available.

## 2 Methods

We carried out a prospectively registered (PROSPERO ID 190939) meta-analysis of randomized placebo-controlled trials of RDN for hypertension in accordance with published guidance [6].

### 2.1 Study selection

We performed a systematic search of MEDLINE and EMBASE databases and the Cochrane Central Register of Controlled trials from 2000 to November 2020 using the search strategy outlined in the Supplementary Appendix. Two independent reviewers performed the search and literature screening (YA and JPH), with disputes resolved by consensus. There were no language restrictions. We also hand-searched the bibliographies of relevant selected studies, reviews and other meta-analyses to identify any further studies. Recent conference abstracts were also searched to identify newly published studies. Abstracts were reviewed for suitability and full-text articles retrieved appropriately.

We included all randomized placebo-controlled studies of RDN for hypertension if they reported either office or 24-hour ambulatory BP changes from baseline. Unblinded studies were not considered as several previous meta-analyses have shown these provide inaccurate estimations of effect size [7].

### 2.2 Data extraction

The primary efficacy endpoint was change in 24-hour ambulatory systolic blood pressure (ASBP). Secondary efficacy endpoints were change in 24-hour ambulatory diastolic blood pressure (ADBP), change in office systolic blood pressure (OSBP), change in office diastolic blood pressure (ODBP), change in day-time ambulatory systolic blood pressure (DSBP), change in day-time ambulatory diastolic blood pressure (DDBP), change in night-time ambulatory systolic blood pressure (NSBP), change in night-time ambulatory diastolic blood pressure (NDBP).

For all blood pressure endpoints, where trials quoted a baseline-adjusted estimate for the effect size using analysis of covariance, this was used. Otherwise, the difference in change in blood pressure from baseline to final value between arms was used.

We extracted the BP endpoint effect sizes from the analysis of covariance, where possible, along with its 95% confidence interval (CI). In trials where analysis of covariance was not available, we extracted the change in BP from baseline to final in both the RDN and control arms, along with their 95% CIs. The longer-term follow-up of RADIANCE adjusted for baseline BP and also medications at 6 months; for the primary analysis, this measure was used, and a sensitivity analysis would be conducted using 2-month data performed off medication. All endpoints were assessed on an intention-to-treat basis.

Three authors independently extracted data from included trials, with discrepancies resolved by consensus.

### 2.3 Data synthesis

We performed a random-effects meta-analysis using the mean difference in effect sizes and their associated standard errors using the restricted maximum likelihood (REML) estimator. Standard errors for the trials were calculated by dividing the difference between the upper and lower 95% CIs by 2× the appropriate normal score (1.96). Interactions between important characteristics that varied across trials were assessed by performing a mixed-effects meta-analysis with the characteristic as a moderator. A meta-analysis was also performed to ascertain any difference between office and ambulatory blood pressure outcomes in trials which reported both, by calculating the mean and its associated standard error for the difference between the two outcomes. The statistical programming language R [8] with the metafor package [9] was used for statistical analyses. Heterogeneity was assessed with the I^2^ statistic [10].

Sensitivity analyses were performed using a fixed effect analysis, as well as a Jackknife sensitivity analysis excluding each trial in turn. We pre-specified first- and second-generation RDN trials as subgroup analyses, with tests for interaction for the primary outcome.

Included studies were assessed for bias using the Cochrane Risk of Bias tool by two authors independently, with disagreements resolved by consensus. Tests for publication bias would not be performed unless the number of studies analyzed exceeded 10 [11].

## 3 Results

**Fig. 1:**
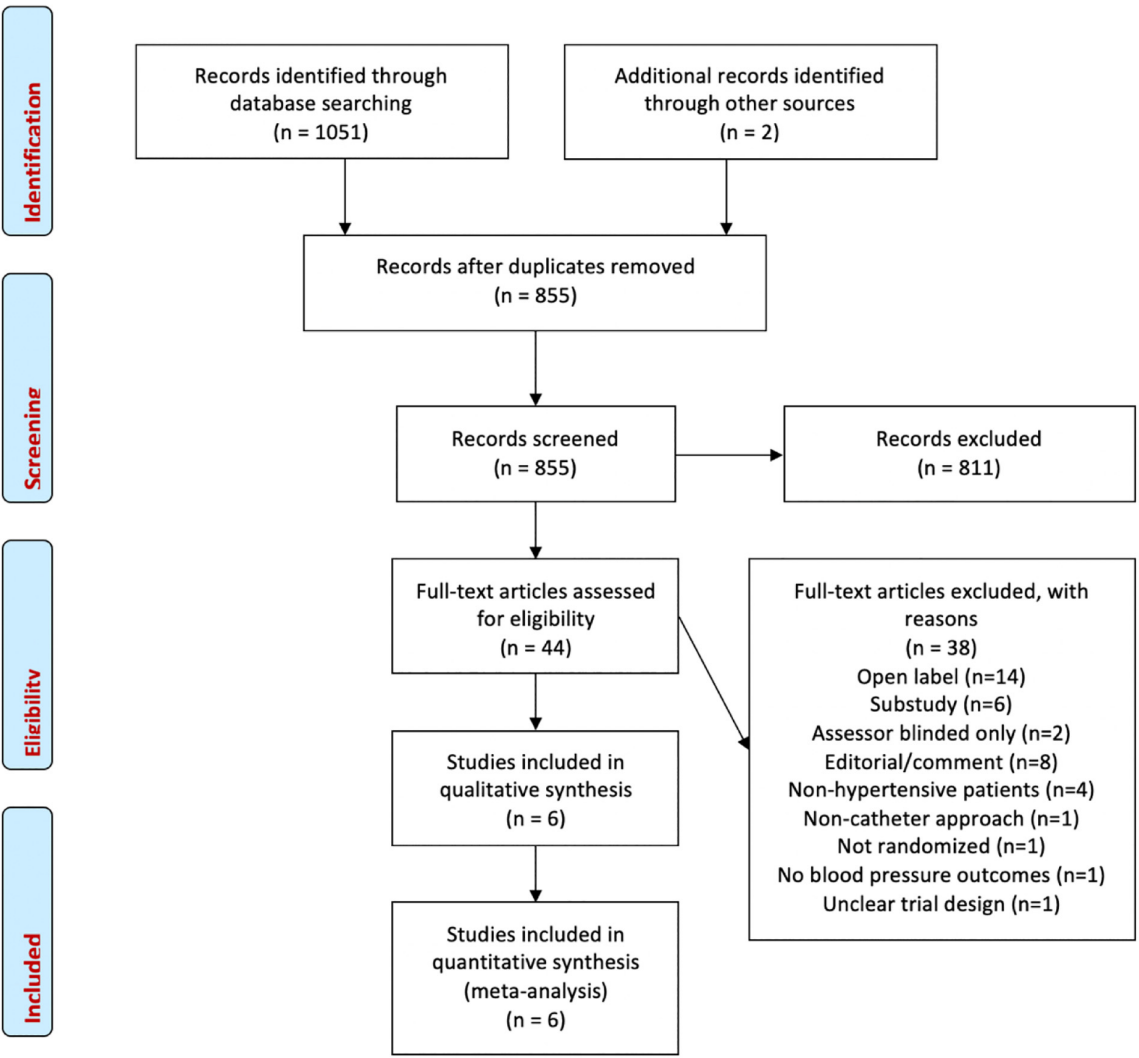
Search strategy and source of included studies.

6 trials [2,[12], [13], [14], [15], [16]], randomizing 1232 patients were eligible for analysis. 713 patients were randomized to RDN and 519 to placebo. The overall weighted mean follow-up duration was 4.86 months. Baseline characteristics are shown in Table 1. The search strategy is shown in Fig. 1.

**Table 1:** Baseline characteristics.

Trial	Year	Device	Follow-up	Number of patients		Baseline OSBP		Baseline ASBP		Age	% Male	% Diabetic	% Non-white
			(months)	Denervation	Placebo	Denervation	Placebo	Denervation	Placebo	(years)			
Symplicity HTN 3	2014	Symplicity	6	364	171	180 (16)	180 (17)	159 (13)	160 (16)	57 (11)	61	45	28
Symplicity FLEX	2015	Symplicity	6	35	36			140 (5)	140 (6)	60 (8)	73	45	0
ReSET	2016	Simplicity	6	36	33	160 (2)	166 (19)	152 (12)	153(13)	56 (9)	74	32	3
SPYRAL HTN OFF MED	2020	Spyral	3	166	165	163 (8)	163 (8)	151 (8)	151 (8)	52 (11)	66	5	26
SPYRAL HTN ON MED	2018	Spyral	6	38	42	165 (7)	164 (8)	152 (7)	151 (7)	53 (10)	84	16	13
RADIANCE-HTN SOLO	2019	Paradise	6	74	72	143 (15)	145 (16)	150 (8)	150 (10)	54 (10)	42	5	23

Continuous data are mean (SD), count data are percentages. *This refers to the number of randomized patients. Further details on the number of patients randomized to each arm for which data were available for each endpoint are detailed within the text of the results. ASBP = Ambulatory systolic blood pressure. OSBP = Office systolic blood pressure.

Risk of bias assessment is shown in Table 2. All trials were judged either moderate-to-high or high-quality.

**Table 2:** Cochrane risk of bias assessment.

Trial	Random sequence generation	Allocation concealment	Blinding of participants & personnel	Blinding of outcome assessment	Incomplete outcome data	Selective reporting	Overall quality
RADIANCE	Low risk Computer-generated permuted blocks.	Low risk Computer-generated permuted blocks accessible only to procedural staff.	Low risk Patients were blinded for full duration as follow-up, facilitated by sham procedure and sedation.	Low Risk Blinded trial staff did at follow-up visits. Adequate blinding by blinding indices.	Low risk 10 patients assigned to renal denervation and 14 assigned to placebo excluded. No unaccounted-for exclusions.	Low risk All endpoints on CT.gov mentioned, but not all reported (NB: these are pre-specified to continue until 36 months so intentionally may not be included in this primary analysis)	High A well conducted, randomized, sham controlled trial of the change in ambulatory BP, analyzed according to ITT
ReSET	Low risk Computer-generated, presumably simple randomization.	Unclear Patient randomized during procedure, but methods unclear.	Low risk Patients were blinded for full duration as follow-up, facilitated by sham procedure and sedation.	Low risk Blinded trial staff did at follow-up visits. Adequate blinding by blinding index.	Low risk 17 patients excluded for unsuitable anatomy and one exclusion for myocardial infarction. No unaccounted-for exclusions.	Low risk All endpoints on CT.gov reported	Moderate-High A moderately-well conducted, randomized, placebo-controlled single-operator trial of the change in ambulatory BP, analyzed according to ITT. Brief data regarding randomization process.
SPYRAL HTN-OFF	Low risk Computer-generated permuted blocks.	Low risk Performing physician blinded to allocation until angiography complete.	Low risk Patients were blinded for full duration as follow-up, facilitated by sham procedure and sedation.	Low risk Blinded trial staff did at follow-up visits. Adequate blinding by blinding index.	Low/moderate risk 6 patients meeting escape criteria and 1 patient missing ABPM at baseline. No unaccounted-for exclusions.	Low risk All endpoints on CT.gov reported	Moderate-High A well conducted placebo-controlled trial of the change in ambulatory BP, analyzed according to ITT
SPYRAL HTN-ON	Low risk Computer-generated permuted blocks	Low risk Performing physician blinded to allocation until angiography complete.	Low risk Patients were blinded for full duration as follow-up, facilitated by sham procedure and sedation.	Low risk Blinded trial staff did at follow-up visits. Adequate blinding by blinding index.	Low risk All exclusions accounted for, with 5 patients meet pre-defined trial ‘escape criteria’. No unaccounted-for exclusions.	Low risk All endpoints on CT.gov reported	High A well conducted placebo-controlled trial of the change in ambulatory BP, analyzed according to ITT
SYMPLICITY FLEX	Low risk Computer-generated simple randomization	Low risk Randomization list managed by an independent IT expert.	Mod risk Sham procedure involving administration of saline but no mention of sedation or blindfolding.	Low/Mod risk All investigators (including personnel responsible for BP assessment) were blinded to treatment assignment. However, no blinding index reported.	Low/Mod risk 3 patients lost to follow-up and 3 excluded from denervation arm. 1 lost to follow-up and 1 excluded from sham arm as did not receive sham procedure due to organizational error. No unaccounted-for exclusions.	Low risk All endpoints on CT.gov reported	Moderate-High A moderately-well conducted placebo-controlled trial of the change in ambulatory BP, analyzed according to ITT. Issues regarding loss to follow up and exclusions noted.
SYMPLICITY HTN 3	Unclear Clearly mentions randomized but no details provided	Unclear Exact randomization procedure unclear.	Low risk Patients were blinded for full duration as follow-up, facilitated by sham procedure and sedation.	Low risk Assessors were blinding and adequate blinding demonstrated by blinding index.	Low/Mod risk 2 patients died and 1 patient withdrew consent in denervation arm. 1 died and 1 withdrew consent in placebo arm. 11 missed 6-month BP measurement, whilst 1 missed 6-month BP measurement in sham arm.	Low risk All endpoints on CT.gov reported	Moderate-High A well conducted placebo-controlled trial of the change in office BP, analyzed according to ITT. Brief data regarding randomization process and numerous missing BP data.

### 3.1 Ambulatory BP

There was no significant heterogeneity in outcome measures unless stated.

**Fig. 2:**
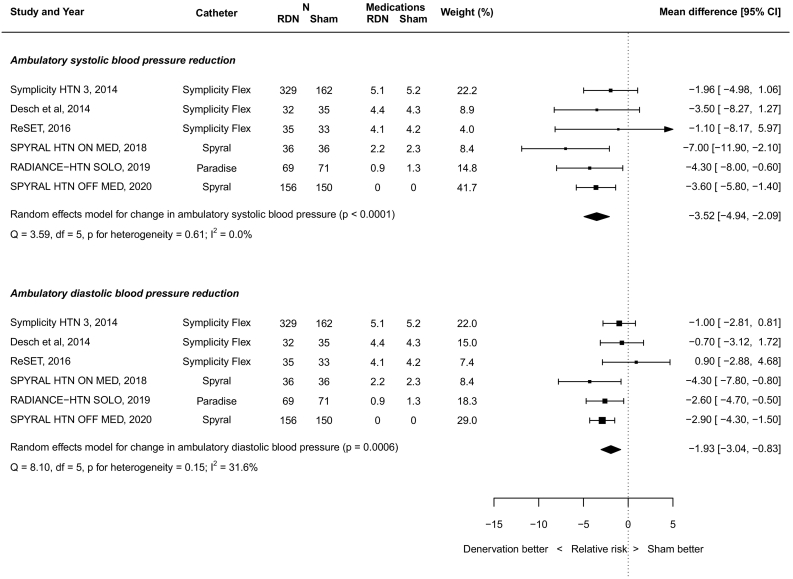
Random-effects meta-analysis of ambulatory blood pressure effect size.

RDN resulted in a significant reduction in ASBP (−3.52 mmHg; 95% CI -4.94 to −2.09; *p* < 0.0001; Fig. 2). RDN also resulted in a significant reduction in ADBP (−1.93 mmHg; 95% CI -3.04 to −0.83, *p* = 0.0006; Fig. 2).

### 3.2 Daytime and nighttime BP

RDN resulted in a significant reduction in DSBP (−3.66 mmHg; 95% CI -5.63 to −1.70; *p* = 0.0003; see Supplementary Appendix).

RDN also resulted in a significant reduction in DDBP (effect size −1.96 mmHg; 95% CI -3.26 to −0.65, *p* = 0.0034; see Supplementary Appendix).

RDN resulted in a significant reduction in NSBP (−3.78 mmHg; 95% CI -6.25 to −1.31; *p* = 0.0027; see Supplementary Appendix).

RDN did not result in a significant reduction in nighttime diastolic blood pressure (−1.57 mmHg; 95% CI -3.41 to 0.28, *p* = 0.0955; see Supplementary Appendix). There was significant heterogeneity (I^2^ = 74.9%).

### 3.3 Office BP

**Fig. 3:**
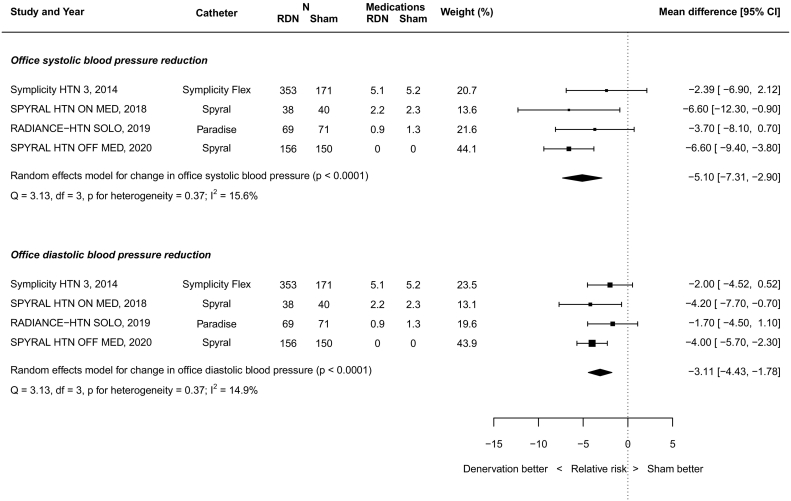
Random-effects meta-analysis of office blood pressure effect size.

RDN resulted in a significant reduction in OSBP (−5.10 mmHg; 95% CI -7.31 to −2.90, *p* < 0.0001; Fig. 3).

RDN also resulted in a significant reduction in ODBP (−3.11 mmHg; 95% CI -4.43 to −1.78, p < 0.0001; Fig. 3).

### 3.4 Safety

Across the 6 trials, there were 3 deaths (2 in the denervation arm and 1 in the control arm; both these occurred in Symplicity HTN 3). There were 4 strokes in the denervation arm and 5 in the control arm. There was one embolism and one vascular complication in the denervation arm (again both in Symplicity HTN 3), as well as 1 case of new renal artery stenosis. 1 patient in the denervation arm required renal artery stenting (in RADIANCE HTN SOLO; this patient had a renal artery stenosis at baseline that was not detected and would have resulted in exclusion from the trial had it been).

### 3.5 Subgroup analyses

There was no significant effect of first versus second generation trials on either ASBP (p for interaction = 0.199) or OSBP (p for interaction = 0.1713). Meta-regression using mixed effects models were used to investigate any significant interaction between trial characteristics and ambulatory systolic blood pressure effect size. There was no significant interaction between the presence of background antihypertensive medications and effect size (difference of −1.10 mmHg for trials off medications; 95% CI -4.40 to −2.2 mmHg; *p* = 0.514).

### 3.6 Sensitivity analyses

All results were consistent when assessed by fixed effect (see Supplementary Appendix). A sensitivity analysis using the initial 2-month off-medication results from RADIANCE was consistent with the primary analysis (see Supplementary Appendix). A full jackknife sensitivity analysis was performed by excluding each individual trial and repeating the meta-analysis for all endpoints. All results were consistent with the primary analyses (see Supplementary Appendix).

## 4 Discussion

RDN successfully lowers BP when measured under blinded placebo-controlled conditions, whether BP is documented in the office or by ambulatory recording. Both SBP and DBP are significantly reduced by RDN. The effect size is completely different in magnitude to that reported in unblinded trials [7].

Our analysis includes the longer-term follow-up of RADIANCE, as well as the full results from SPYRAL HTN OFF MED; the latter trial represents 27% of the total placebo-controlled trial data. Prior meta-analytic work has claimed that second generation catheters are effective in reducing BP, whereas first generation devices are not [17]. Our analysis demonstrates this is not the case. All trials showed a statistically similar effect size. The way to recognize this is to formally assess for heterogeneity between trial results, and not to dichotomize trials into positive and negative because doing so discards the information contained in the confidence intervals. Specifically, this analysis shows Symplicity HTN-3 is perfectly compatible with all other trials. Furthermore, subgroup analyses for first generation versus second generation trials did not find evidence of a statistically significant impact on the primary endpoint.

Early research in the field, reporting large office blood pressure reductions (~30 mmHg) and much smaller ambulatory blood pressure reductions (~10 mmHg), was interpreted as genuine [18] and evidence that renal denervation had a specific additional effect on alerting responses. In fact, this appears not to be correct. The present analysis shows that the reduction in office blood pressure is no different from the reduction in ambulatory blood pressure (p = NS for difference between effects). Renal denervation therefore shows the same phenomenon as antihypertensive medication. When documented by unblinded staff, office blood pressure falls more than ambulatory; when documented by blinded staff, office blood pressure falls by the same amount as ambulatory [1].

Based on trial data of antihypertensive drugs, an effect size of 5 mmHg on OSBP persisting in the long term should confer 10% reduction in major adverse cardiac events and 7.5% reduction in all-cause mortality [19]. It is not known whether the effect size of RDN varies in the long term. For example, in SPYRAL HTN-ON MED, the difference between arms was not significant at 3 months, but was significant at 6 months [15]. Adherence to medication is lower in real-life than in clinical trials, and therefore the benefits of a single procedure with an ‘always on’ effect may be greater in the long-term than that seen with drug-therapy. Additionally, patients considering renal denervation are often those most adverse to taking addition or even any medications. In SPYRAL HTN-ON MED for example, over 35% of participants were nonadherent to their antihypertensive medications.

This meta-analysis also indicates that the effect size of renal denervation is consistent regardless of whether it is used in patients who have not yet started medications or in patients who are already established on medications but have inadequate control. This suggests it could be used at several points within the overall strategy of hypertension management.

Renal denervation seems to have a reasonable safety profile. Major adverse events were rare, and were no more common than following a placebo procedure.

### 4.1 Limitations

All trials in this analysis report results between 2 and 6 months from RDN, so there is currently no bias-resistant evidence of what happens to the effect size after this. Safety events are relatively rare after RDN and therefore this analysis cannot exclude a low rate of excess events with RDN over placebo.

## 5 Conclusions

The totality of blinded, randomized placebo-controlled data shows RDN is safe and provides genuine reduction in BP for at least 6 months post-procedure. If this effect continues long term, RDN might provide a life-long 10% relative risk reduction in major adverse cardiac events and 7.5% relative risk reduction in all-cause mortality.

## CRediT authorship contribution statement

**Yousif Ahmad**: Conceptualization, Methodology, Software, Formal analysis, Data Curation, Writing - Original Draft, Writing - Review & Editing, Visualization, Supervision.

**Christopher Kane**: Data Curation, Investigation, Writing – Review & Editing.

**Ahran D Arnold**: Data Curation, Investigation, Writing – Review & Editing.

**Christopher M Cook**: Data Curation, Investigation, Writing – Review & Editing.

**Daniel Keene**: Data Curation, Investigation, Writing – Review & Editing.

**Matthew Shun**-**Shin**: Data Curation, Investigation, Writing – Review & Editing.

**Graham Cole**: Data Curation, Investigation, Writing – Review & Editing.

**Rasha Al**-**Lamee**: Data Curation, Investigation, Writing – Review & Editing.

**Darrel P Francis**: Writing – Original Draft, Writing – Review & Editing, Supervision.

**James P**. **Howard**: Conceptualization, Methodology, Software, Formal analysis, Data Curation, Writing - Original Draft, Writing - Review & Editing, Visualization, Supervision.

## Declaration of competing interest

All authors have nothing to declare.

## Appendix A Supplementary data

Supplementary material Image 1
