# Supplementary material for: Randomized Blinded Placebo-Controlled Trials of Renal Sympathetic Denervation for Hypertension: A Meta-Analysis
Source: Cardiovasc Revasc Med. 2022 Jan;34:112–8. doi: 10.1016/j.carrev.2021.01.031 (PMC8813172; doi:10.1016/j.carrev.2021.01.031)
Supplement: Supplementary file 1 — Supplementary material [file mmc1.docx]

**Supplementary Appendix**

**Renal sympathetic denervation for hypertension: an updated meta-analysis of 6 randomised blinded sham-controlled trials**

Appendix 1. Search strategy. Page 2

Figure 1. Random-effects meta-analysis of daytime SBP Page 8

Figure 2. Random-effects meta-analysis of daytime DBP Page9

Figure 3. Random-effects meta-analysis of nightime SBP Page 10

Figure 4. Random-effects meta-analysis of nightime DBP Page 11

Figures 5-12. Fixed effect analyses Pages 12-19

Figures 13-20 Sensitivity anaylses using 2-month off med RADIANCE results Pages 20-27

Figure 21-68 Sensitivity Jackknife analyses excluding each trial in turn Pages 28-75

# Search strategy

## OVID

Wolters Kluwer’s Ovid ® was used to search MEDLINE and EMBASE (from January 2010 to present) using the following search strategy on 29^th^ May 2020:

1. "renal denervation" or "renal sympathetic denervation"
2. AND “trial”
3. AND “Randomised” OR “Randomized”

References of all included studies, and review/systematic review/meta-analysis articles identified in the search, were hand searched for additional references. This did not yield any additional results.

## The Cochrane Central Register of Controlled Trials

The Cochrane Central Register of Controlled Trials was searched (from January 2010 to present) using the following search strategy on 29^th^ May 2020:

1. "renal denervation" or "renal sympathetic denervation"
2. AND “trial”
3. AND “Randomised” OR “Randomized”

References of all included studies, and review/systematic review/meta-analysis articles identified in the search, were hand searched for additional references. This did not yield any additional resul

Figure 1. Random-effects meta-analysis of daytime systolic blood pressure effect size.

Figure 2. Random-effects meta-analysis of daytime diastolic blood pressure effect size.

Figure 3. Random-effects meta-analysis of nighttime systolic blood pressure effect size.

Figure 4. Random-effects meta-analysis of nighttime diastolic blood pressure effect size.

Figure 5. Fixed-effect meta-analysis of ambulatory systolic blood pressure effect size.

Figure 6. Fixed-effect meta-analysis of ambulatory diastolic blood pressure effect size.

Figure 7. Fixed-effect meta-analysis of office systolic blood pressure effect size.

Figure 8. Fixed-effect meta-analysis of office diastolic blood pressure effect size.

Figure 9. Fixed-effect meta-analysis of daytime systolic blood pressure effect size.

Figure 10. Fixed-effect meta-analysis of daytime diastolic blood pressure effect size.

Figure 11. Fixed-effect meta-analysis of nighttime systolic blood pressure effect size.

Figure 12. Fixed-effect meta-analysis of nighttime diastolic blood pressure effect size.

Figure 13. Sensitivity anaylsis using 2-month off med RADIANCE results for ambulatory systolic blood pressure effect size.

Figure 14. Sensitivity anaylsis using 2-month off med RADIANCE results for ambulatory diastolic blood pressure effect size.

Figure 15. Sensitivity anaylsis using 2-month off med RADIANCE results for office systolic blood pressure effect size.

Figure 16. Sensitivity anaylsis using 2-month off med RADIANCE results for office diastolic blood pressure effect size.

Figure 17. Sensitivity anaylsis using 2-month off med RADIANCE results for daytime systolic blood pressure effect size.

Figure 18. Sensitivity anaylsis using 2-month off med RADIANCE results for daytime diastolic blood pressure effect size.

Figure 19. Sensitivity anaylsis using 2-month off med RADIANCE results for nighttime systolic blood pressure effect size.

Figure 20. Sensitivity anaylsis using 2-month off med RADIANCE results for nighttime diastolic blood pressure effect size.

Figure 21. Sensitivity anaylsis excluding SPYRAL HTN OFF MED for ambulatory systolic blood pressure effect size.

Figure 22. Sensitivity anaylsis excluding SPYRAL HTN OFF MED for ambulatory diastolic blood pressure effect size.

Figure 23. Sensitivity anaylsis excluding SPYRAL HTN OFF MED for office systolic blood pressure effect size.

Figure 24. Sensitivity anaylsis excluding SPYRAL HTN OFF MED for office diastolic blood pressure effect size.

Figure 25. Sensitivity anaylsis excluding SPYRAL HTN OFF MED for daytime systolic blood pressure effect size.

Figure 26. Sensitivity anaylsis excluding SPYRAL HTN OFF MED for daytime diastolic blood pressure effect size.

Figure 27. Sensitivity anaylsis excluding SPYRAL HTN OFF MED for results for nighttime systolic blood pressure effect size.

Figure 28. Sensitivity anaylsis excluding SPYRAL HTN OFF MED for nighttime diastolic blood pressure effect size.

Figure 29. Sensitivity anaylsis excluding SPYRAL HTN ON MED for ambulatory systolic blood pressure effect size.

Figure 30. Sensitivity anaylsis excluding SPYRAL HTN ON MED for ambulatory diastolic blood pressure effect size.

Figure 31. Sensitivity anaylsis excluding SPYRAL HTN ON MED for office systolic blood pressure effect size.

Figure 32. Sensitivity anaylsis excluding SPYRAL HTN ON MED for office diastolic blood pressure effect size.

Figure 33. Sensitivity anaylsis excluding SPYRAL HTN ON MED for daytime systolic blood pressure effect size.

Figure 34. Sensitivity anaylsis excluding SPYRAL HTN ON MED for daytime diastolic blood pressure effect size.

Figure 35. Sensitivity anaylsis excluding SPYRAL HTN ON MED for results for nighttime systolic blood pressure effect size.

Figure 36. Sensitivity anaylsis excluding SPYRAL HTN ON MED for nighttime diastolic blood pressure effect size.

Figure 37. Sensitivity anaylsis excluding RADIANCE for ambulatory systolic blood pressure effect size.

Figure 38. Sensitivity anaylsis excluding RADIANCE for ambulatory diastolic blood pressure effect size.

Figure 39. Sensitivity anaylsis excluding RADIANCE for office systolic blood pressure effect size.

Figure 40. Sensitivity anaylsis excluding RADIANCE for office diastolic blood pressure effect size.

Figure 41. Sensitivity anaylsis excluding RADIANCE for daytime systolic blood pressure effect size.

Figure 42. Sensitivity anaylsis excluding RADIANCE for daytime diastolic blood pressure effect size.

Figure 43. Sensitivity anaylsis excluding RADIANCE for results for nighttime systolic blood pressure effect size.

Figure 44. Sensitivity anaylsis excluding RADIANCE for nighttime diastolic blood pressure effect size.

Figure 45. Sensitivity anaylsis excluding ReSET for ambulatory systolic blood pressure effect size.

Figure 46. Sensitivity anaylsis excluding ReSET for ambulatory diastolic blood pressure effect size.

Figure 47. Sensitivity anaylsis excluding ReSET for office systolic blood pressure effect size.

Figure 48. Sensitivity anaylsis excluding ReSET for office diastolic blood pressure effect size.

Figure 49. Sensitivity anaylsis excluding ReSET for daytime systolic blood pressure effect size.

Figure 50. Sensitivity anaylsis excluding ReSET for daytime diastolic blood pressure effect size.

Figure 51. Sensitivity anaylsis excluding ReSET for results for nighttime systolic blood pressure effect size.

Figure 52. Sensitivity anaylsis excluding ReSET for nighttime diastolic blood pressure effect size.

Figure 53. Sensitivity anaylsis excluding SYMPLICITY Flex for ambulatory systolic blood pressure effect size.

Figure 54. Sensitivity anaylsis excluding SYMPLICITY Flex for ambulatory diastolic blood pressure effect size.

Figure 55. Sensitivity anaylsis excluding SYMPLICITY Flex for office systolic blood pressure effect size.

Figure 56. Sensitivity anaylsis excluding SYMPLICITY Flex for office diastolic blood pressure effect size.

Figure 57. Sensitivity anaylsis excluding SYMPLICITY Flex for daytime systolic blood pressure effect size.

Figure 58. Sensitivity anaylsis excluding SYMPLICITY Flex for daytime diastolic blood pressure effect size.

Figure 59. Sensitivity anaylsis excluding SYMPLICITY Flex for results for nighttime systolic blood pressure effect size.

Figure 60. Sensitivity anaylsis excluding SYMPLICITY Flex for nighttime diastolic blood pressure effect size.

Figure 61. Sensitivity anaylsis excluding SYMPLICITY HTN 3 for ambulatory systolic blood pressure effect size.

Figure 62. Sensitivity anaylsis excluding SYMPLICITY HTN 3 for ambulatory diastolic blood pressure effect size.

Figure 63. Sensitivity anaylsis excluding SYMPLICITY HTN 3 for office systolic blood pressure effect size.

Figure 64. Sensitivity anaylsis excluding SYMPLICITY HTN 3 for office diastolic blood pressure effect size.

Figure 65. Sensitivity anaylsis excluding SYMPLICITY HTN 3 for daytime systolic blood pressure effect size.

Figure 66. Sensitivity anaylsis excluding SYMPLICITY HTN 3 for daytime diastolic blood pressure effect size.

Figure 67. Sensitivity anaylsis excluding SYMPLICITY HTN 3 for results for nighttime systolic blood pressure effect size.

Figure 68. Sensitivity anaylsis excluding SYMPLICITY HTN 3 for nighttime diastolic blood pressure effect size.
